# Supplementary material for: Impact of Processing Parameters on the Quality of Pharmaceutical Solid Dosage Forms Produced by Fused Deposition Modeling (FDM)
Source: Pharmaceutics. 2019 Nov 27;11(12):633. doi: 10.3390/pharmaceutics11120633 (PMC6956065; doi:10.3390/pharmaceutics11120633)
Supplement: Supplementary file 1 [file pharmaceutics-11-00633-s001.pdf]

pharmaceutics

MDPI

Supplementary Materials: Impacts of processing parameters on the precision of Fused Deposition Modelling (FDM) 3D printing for pharmaceutical dosage form fabrication

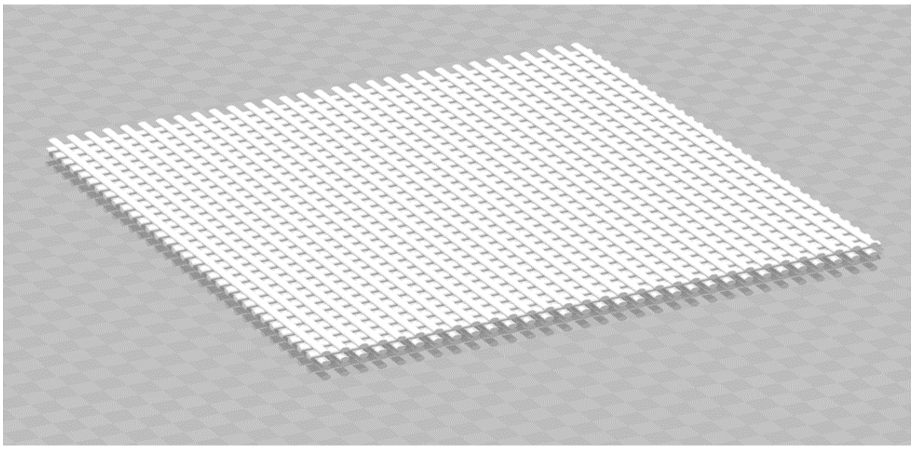

Figure S1. Computer-generated image of the STL file design for the porous film.

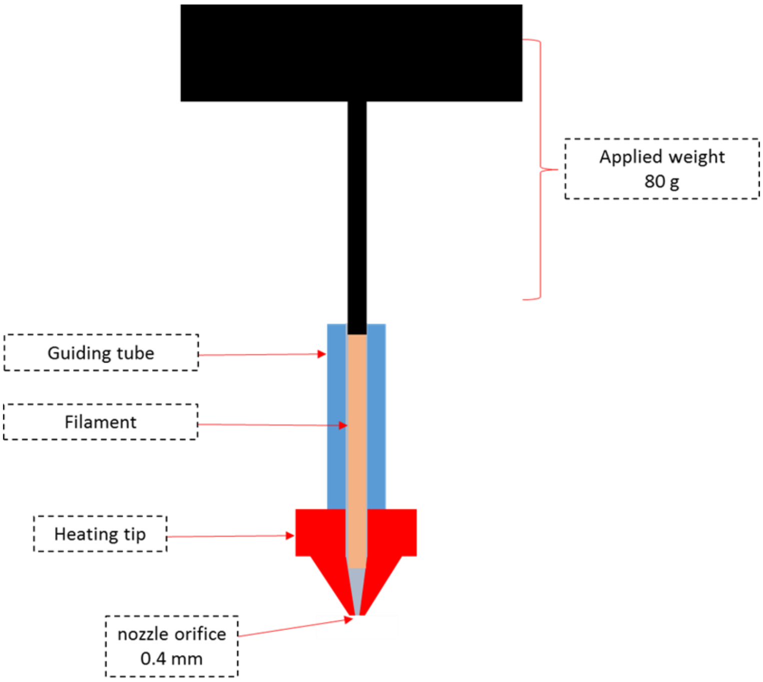

Figure S2. Illustration for the method adapted for measuring the melt flow index (MFI) for different materials.

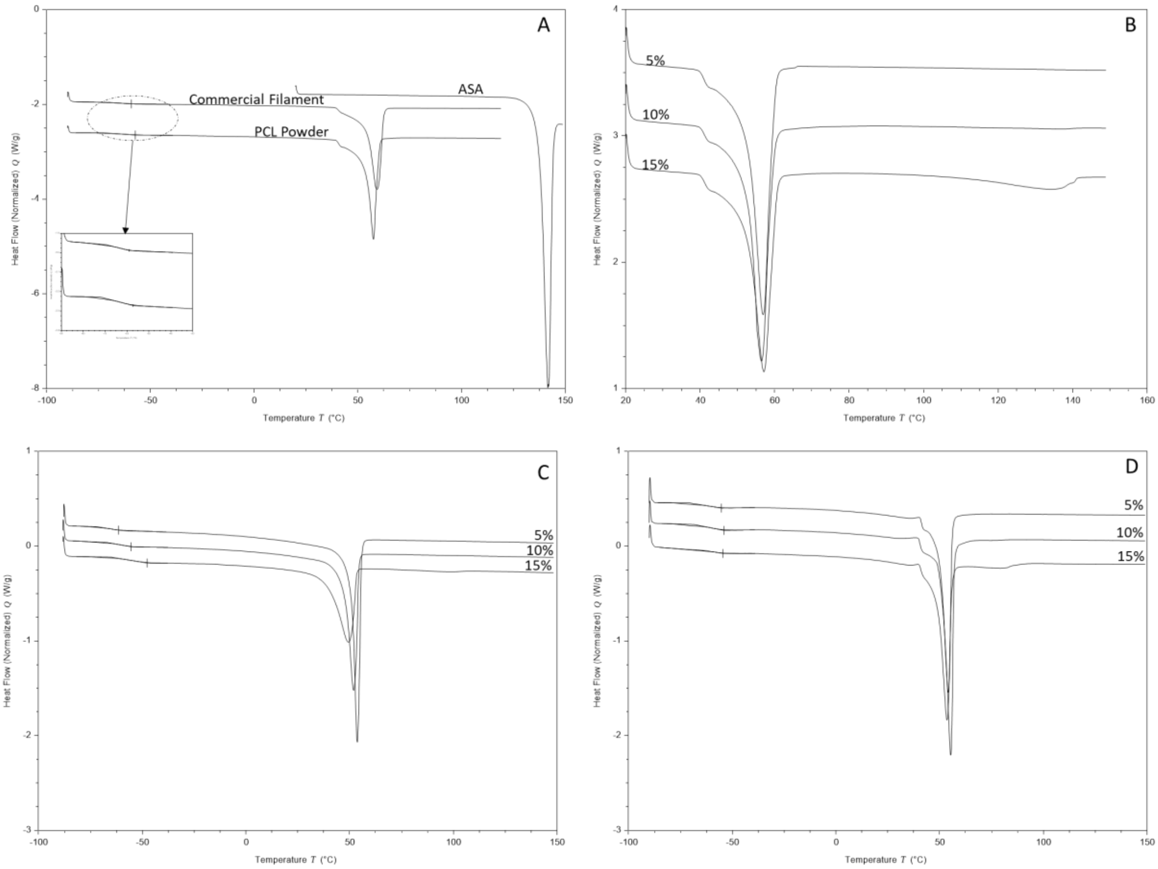

Figure S3. DSC thermograms of **A**) Raw materials. **B**) ASA-PCL powder mixtures (first heating cycle). **C**) ASA-PCL powder mixtures (second heating cycle). **D**) HME fabricated filaments**.**

The DSC thermograms (Figure S3) of the ASA-PCL physical mixtures suggest that the mechanism by which ASA is incorporated into PCL is via thermal dissolution. This is evidenced by the absence of the melting of ASA in both the 5% and 10% physical mixtures during the first heating cycle, but the existence of a significant difference in both the T_g and the T_m of PCL between the formulations in the second cycle. The depressed T_m of ASA was seen in the 15% physical mix at approximately 135 °C, but was not seen in the second cycle. ASA appears to have an antiplasticization effect on PCL, as evidenced by the increase in the T_g of PCL per ASA concentration in the physical mix. The T_g of ASA was presumably seen at -33 °C during the second heating cycle. However, it should be noted that, due to ASA rapidly degrading beyond its melting point, it is difficult to ascertain whether the detected transition actually belongs to ASA or to a related compound to which ASA degrades.

The thermogram of the 5% formulation showed no signs of crystalline ASA within the matrix. Furthermore, the ATR-FTIR spectrum (Figure 6 in the main manuscript) of the 5% filament showed a clear shift in the C=O stretching band of PCL from 1725 cm^{-1} to 1722 cm^{-1}, and the C-OH peak shifted from 1163 cm^{-1} to 1165 cm^{-1}. Furthermore, the C-O stretch at 1293 cm^{-1} was notably a weak singlet peak, unlike the strong doublet seen in crystalline ASA. The absence of peaks characteristic to crystalline ASA in the ATR-FTIR spectrum, coupled with the absence of the T_m of ASA in the thermogram strongly suggests ASA is molecularly dispersed in the PCL matrix. The shift in the C=O and C-OH peaks of PCL point towards hydrogen bonding at the carboxyl groups as the main ASA-PCL interaction maintaining the solid dispersion.

The endothermic event seen at 80 °C in the thermogram of the 15% filament likely corresponds to the depressed melting endotherm of ASA. The absence of any significant difference in the glass transition and melting endotherm of PCL in the 10% and the 15% formulations further supports that the continuous phases of the two formulations are identical, indicating that the thermal event seen at

80 °C is crystallized ASA. The ATR-FTIR spectra (Figure 5 in the main manuscript) of the 10% and 15% filaments both clearly display the C-O doublet previously seen in the spectrum of crystalline ASA. However, the peak at 1290 cm^{-1} has shifted to 1295 cm^{-1} in the 10% filament, and to 1294 cm^{-1} in the 15% formulation. The C-OH peak seen in crystalline ASA at 1184 cm^{-1} was seen in the 10% peak at 1182 cm^{-1}. This indicates that both the 10% and 15% formulation contain a crystalline fraction of ASA, which was not seen in the 10% thermogram possibly due to detection limits of the DSC. The shifted peaks of the C-O and C-OH groups still indicate that the molecularly dispersed fraction of ASA is maintained by hydrogen bonding at the carboxyl groups. The saturation solubility of ASA in PCL is likely somewhere between 5% and 10%.

Table 1. Microscopic images depicting road width measurements. N.B. images may be resized for viewing the full details of the measurements.

**30 mm/sec**			
**temperature**	Top Layer	Middle layer	Platform layer
**90 °C**	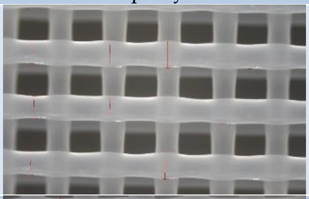	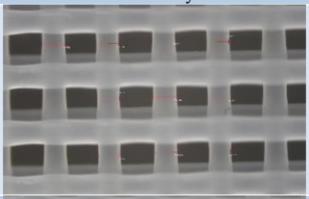	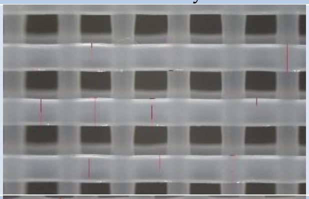
**100 °C**	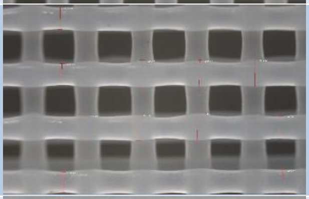	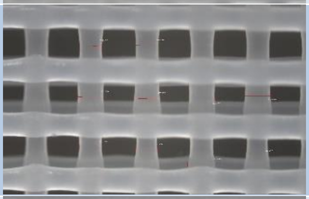	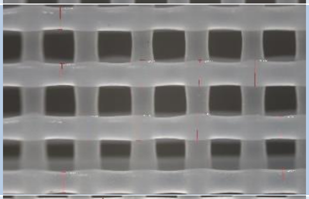
**110 °C**	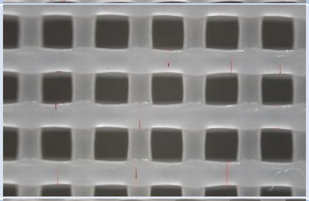	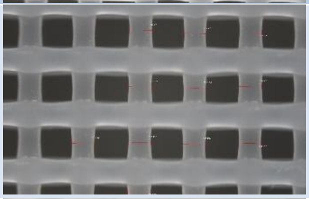	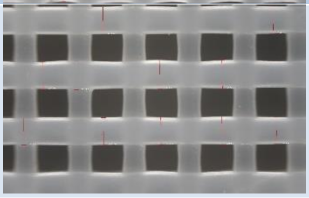
**120 °C**	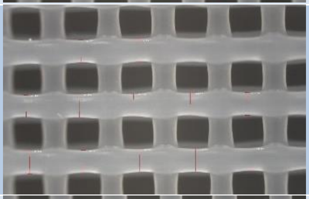	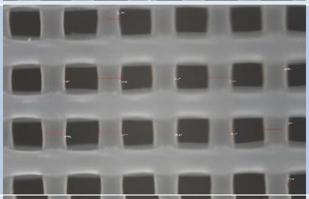	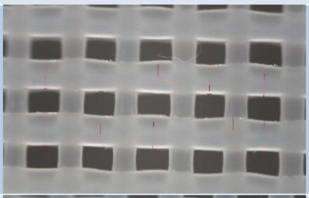
**130 °C**	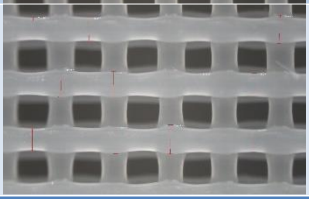	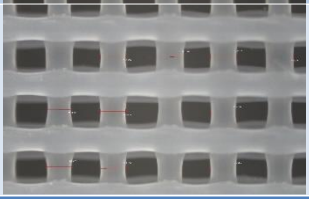	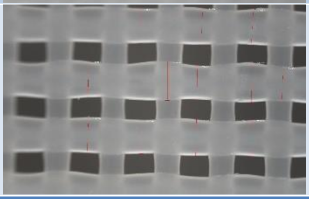
**90 mm/sec**			
**90 °C**	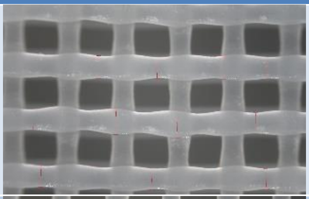	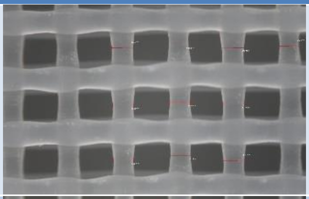	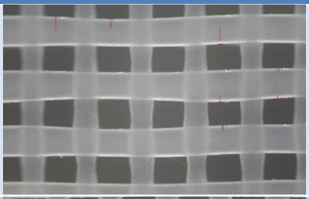
**100 °C**	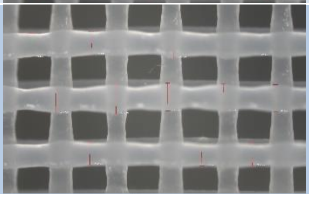	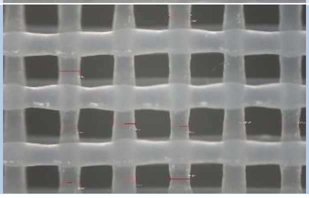	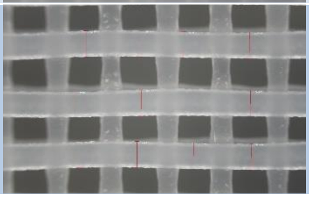

**110 °C**	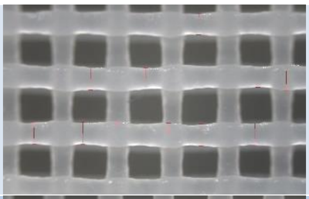	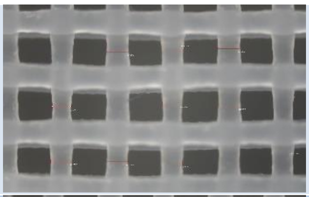	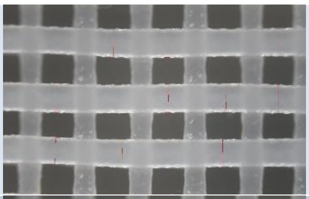
**120 °C**	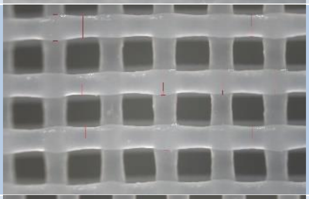	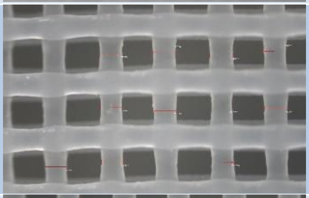	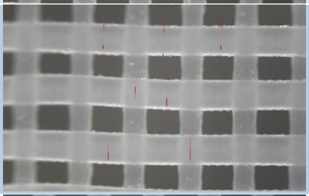
**130 °C**	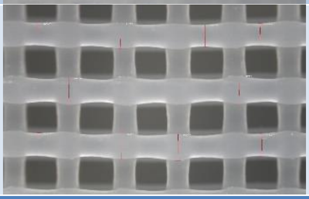	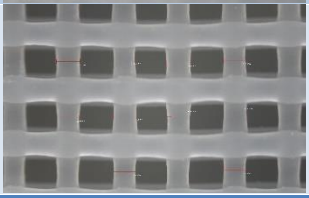	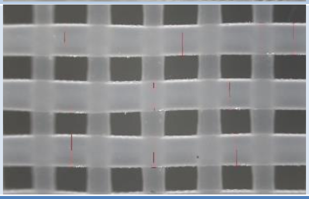
**160 mm/sec**			
**90 °C**	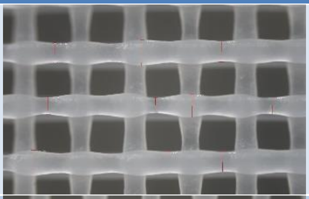	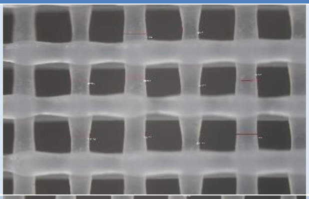	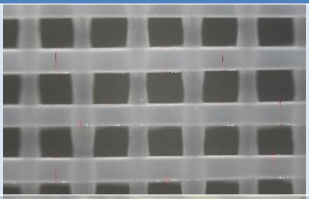
**100 °C**	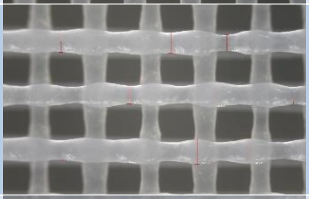	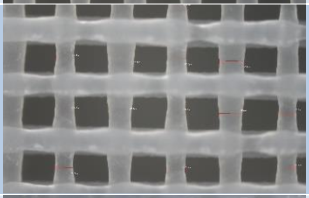	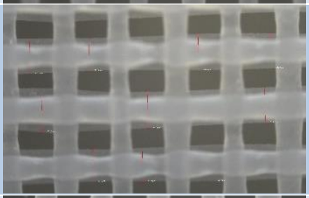
**110 °C**	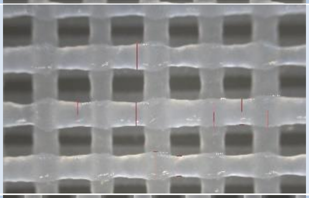	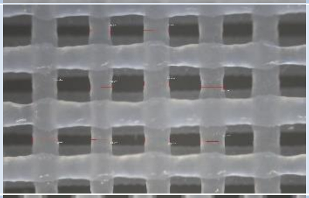	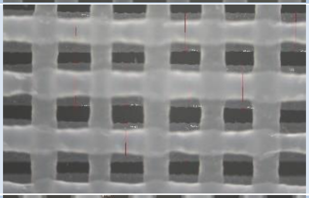
**120 °C**	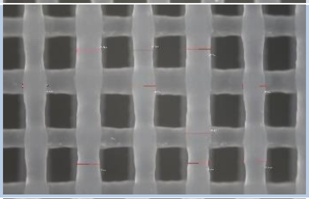	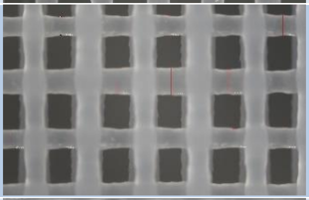	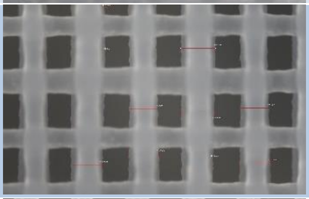
**130 °C**	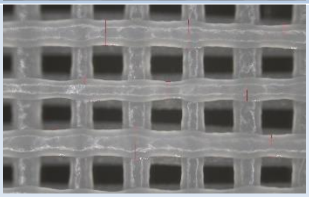	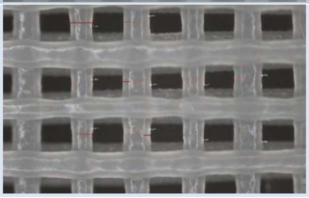	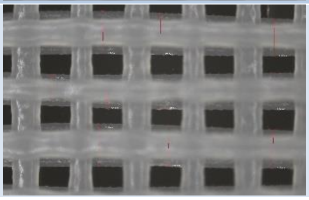
